# Breast cancer evaluation by fluorescent dot detection using combined mathematical morphology and multifractal techniques

**DOI:** 10.1186/1746-1596-6-S1-S21

**Published:** 2011-03-30

**Authors:** Branimir Reljin, Milorad Paskas, Irini Reljin, Korski Konstanty

**Affiliations:** 1 Digital Image Processing, Telemedicine and Multimedia Lab, Faculty of Electrical Engineering, University of Belgrade, Belgrade, Serbia; 2 Molecular Biology Lab, Department of Pathology, Wielkoposka Cancer Center, Poznan, Poland

## Abstract

**Background:**

Fluorescence *in situ* hybridization (FISH) is very accurate method for measuring HER2 gene copies, as a sign of potential breast cancer. This method requires small tissue samples, and has a high sensitivity to detect abnormalities from a histological section. By using multiple colors, this method allows the detection of multiple targets simultaneously. The target parts in the cells become visible as colored dots. The HER-2 probes are visible as orange stained spots under a fluorescent microscope while probes for centromere 17 (CEP-17), the chromosome on which the gene HER-2/neu is located, are visible as green spots.

**Methods:**

The conventional analysis involves the scoring of the ratio of HER-2/neu over CEP 17 dots within each cell nucleus and then averaging the scores for a number of 60 cells. A ratio of 2.0 of HER-2/neu to CEP 17 copy number denotes amplification. Several methods have been proposed for the detection and automated evaluation (dot counting) of FISH signals. In this paper the combined method based on the mathematical morphology (MM) and inverse multifractal (IMF) analysis is suggested. Similar method was applied recently in detection of microcalcifications in digital mammograms, and was very successful.

**Results:**

The combined MM using top-hat and bottom-hat filters, and the IMF method was applied to FISH images from Molecular Biology Lab, Department of Pathology, Wielkoposka Cancer Center, Poznan. Initial results indicate that this method can be applied to FISH images for the evaluation of HER2/neu status.

**Conclusions:**

Mathematical morphology and multifractal approach are used for colored dot detection and counting in FISH images. Initial results derived on clinical cases are promising. Note that the overlapping of colored dots, particularly red/orange dots, needs additional improvements in post-processing.

## Background

Breast cancer is the most common cancer for women worldwide, comprising 16% of all female cancers. From reports of the World Health Organization (WHO) [1], more than one million cases of breast cancer are diagnosed every year, mainly (about 75 percent) for women aged 50 and older. Since breast cancer typically spreads from the breast to lymph nodes and then to distant parts of the body (metastasis), very often such spreading can occur prior to the detection of primary cancer. Breast cancer survival rates vary greatly worldwide, ranging from 80% or over in developed countries (North America, Sweden and Japan) to around 60% in middle-income countries and below 40% in low-income countries [2]. The survival is greatly improved if the breast anomalies are detected at early stages through breast self exams and/or mammography [1].

In the late 1980s, researchers discovered that approximately 20 percent of women with breast cancer produce abnormally high amounts of a protein called human epidermal growth factor receptor 2 (HER2), also known as HER2/neu, a member of the HER family of receptor proteins. This protein sends signals to human cells, affecting to their growth and differentiation. A healthy breast cell has 2 copies of the HER2 gene. Some kinds of breast cancer get started when a breast cell has more than 2 copies of that gene (this process is called the *amplification*), and those copies start over-producing the HER2 protein. As a result, the affected cells grow and divide much too quickly [3].

Amplification of HER2 is associated with a poor prognosis (higher rate of recurrence and mortality) and is usually associated with resistance to endocrine therapies. Fortunately, after more decade of research, a drug Herceptin (generic name *trastuzmab*) was discovered and approved for use by the U.S. Food and Drug Administration (FDA) in September 1998, which can be very successful in treatment of advanced breast cancer just for women whose breast cancer cells carry extra copies of a HER2. This drug, given intravenously, once every 2-3 weeks, targets the HER2 protein production. This helps to stop the growth of the HER2 positive cancer cells. Herceptin has shown great promise in increasing patient survival time and reducing the number of deaths from advanced breast cancer. Clinical trials are also investigating whether Herceptin is helpful for women with early-stage breast cancers [4], [5].

Accurate determination of Her-2/neu status in breast carcinoma is essential for therapy planning. Nowadays, the two most widely technologies for the evaluation of HER2 status are immunohistochemistry (IHC) and fluorescence *in situ* hybridization (FISH). The IHC measures the expression of the HER2 protein on the surface of the tumor cell while FISH measures the amplification of the HER2/neu gene present in the cells. The IHC uses low-cost standard microscope observation [6] but this method is more subjective, whereas FISH technique needs more specialized equipment than immunohistochemistry does, but is more objective and permits even automated evaluation [7], [8].

Fluorescence is a physical property of some materials, based on the quantum transitions. When illuminated at specific wavelengths (energy), such materials are excited and, in relaxation phase, emit light, usually at lower wavelengths (lower energy). Different fluorescent markers have been developed for detection specific cells, parts of cells, nuclei and chromosomes. For instance, nuclei can be detected after the treatment with diamidinophenylindole (DAPI), a fluorescent marker that emits blue light [9]. The FDA approved PathVision Her2 FISH kit (Vysis, Downers Grove, USA) uses DNA probes, which are small segments of actual DNA material. When applied to a tumor tissue sample, these DNA probes target the HER-2/neu gene and attach themselves to their target sequence. This process is called *hybridization*. The probes carry special fluorescent markers that emit light, when the probes bind to the HER-2 genes. The HER-2 probes are visible as orange stained spots under a fluorescent microscope. Similarly, probes for centromere 17 (CEP-17), the chromosome on which the gene HER-2/neu is located, are visible as green spots.

Fluorescence microscopy differs from classic optic microscopy. In classic microscopy, the sample to visualize is placed between a source of visible light and the observer. The light is either reflected from the sample (as in mineralogy) or transmitted through the thin tissue (as in cytology). Fluorescence microscopy uses some indirect method, as follows. A high-intensity UV light is directed at the sample; the fluorescent markers in use emit light at specific visible wavelengths; these signals are then observed through adapted filters. The fluorescent signals obtained are recorded on a charge-coupled device (CCD) camera, resulting in as many grayscale images as the number of filters used.

The process of evaluating HER-2/neu status from FISH images involves the manual counting of signals in interphase nuclei which become visible as colored dots. The conventional analysis involves the scoring of the ratio of HER-2/neu over CEP 17 dots within each cell nucleus and then averaging the scores for a number of cells. Several images usually need to be read to reach the desired number of dot-including nuclei. A ratio of HER-2/neu to CEP 17 copy number greater than 2.2 denotes amplification, the ratio less than 1.8 denotes normal state, while results between 1.8 and 2.2 are suspicious and need additional investigations [10].

## Automated evaluation of HER2/neu status

Manual evaluation of HER2/neu status from FISH images may be difficult task since dot counting over a large number of nuclei and over different tissue samples is time consuming and tiresome procedure. Moreover, this procedure needs skilled person for this imaging technique. Nowadays, the equipment for analysis of FISH images permits some kind of semi-automatic analysis with the aid of image processing software, which can display different color channels and apply thresholds for nuclei segmentation. But, the dot counting still remains a difficult procedure for a physician, because nuclei can be poorly segmented, overlapped or clustered [11].

Several systems for automatic detection and evaluation of FISH images are reported [12], [13]. These systems usually use different color channels from RGB image for segmenting and extracting nuclei (in blue channel) and then detecting and counting red/orange (HER2/neu) and green (CEP 17) dots within the segmented nuclei. Although the procedure seems to be easy, in practice a lot of difficulties arise: one of the most severe task is to detect, segment and extract just nuclei. Very good approach is reported in [14] where the author described deep analysis of the structure of FISH images and suggested the novel segmentation method permitting very high accuracy of more than 99% on the dataset containing about 14,000 samples.

## Methods

Detecting relatively small bright dots within the digital image was under the investigation of our recent research targeted to the detection of microcalcifications in digital (or digitized) mammograms [15-17]. By using combined mathematical morphology and multifractal methods we obtained very good detection of microcalcifications even in the case of radiology hard cases – when the breast tissue was very dense [17]. In this paper we will apply similar procedure for detecting and counting fluorescent dots in FISH images.

**Figure 1:**
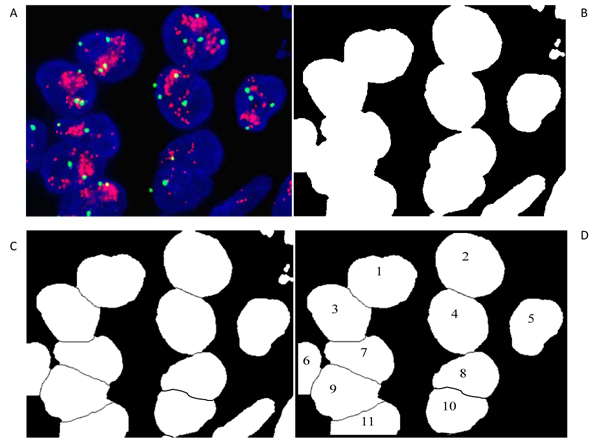
Illustration of the process of nuclei segmentation and indexing.

**Figure 2:**
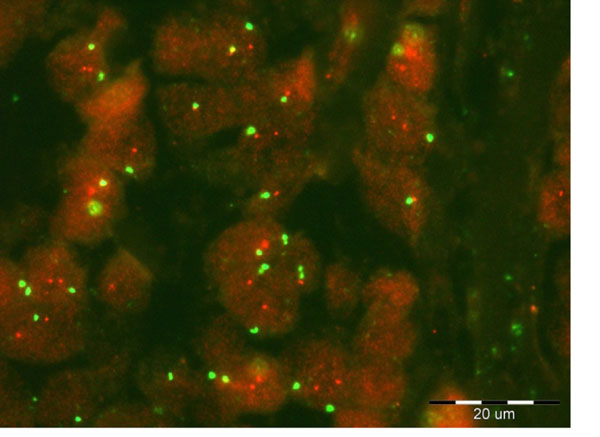
Case 5 of analyzed FISH images. Image without DAPI marker.

The assumption in our procedure is to have FISH images treated with the PathVision Her2 FISH kit. Typical example is depicted in Fig. 1-a. The first step is to segment parts which can be nuclei from the blue channel of the RGB image, Fig. 1-b. Overlapped parts are separated by applying watershed procedure, Fig. 1-c. Selected objects as in Fig. 2-c are analyzed regarding to their shape and size, and objects which are not nuclei are removed, and nuclei are indexed, Fig. 1-d. After that, the colored dots within segmented nuclei are detected and counted. Since colored dots are displayed as bright dots in monochrome images, dots detection was performed by using the two methods similar as in our previous work when detecting microcalcifications [17].

First method is based on mathematical morphology (MM). Instead of applying only top-hat (*TH*) filter, as usually used for extracting bright spots, we suggested the combination of top-hat and bottom-hat (*BH*) filters. The top-hat is defined as the difference of the original image, *I*, and its opening, while the bottom-hat is defined as the image closing minus the original image. In this way, the *TH* filter is an excellent tool for enhancing small bright details from a nonuniform background. Consequently, the *BH* filter produces an opposite effect: one can extract dark features from a brighter background. Note that both filters also equalize a nonuniform background illumination. Moreover, local contrast enhancement with high suppression of surrounding texture can be achieved by adding the difference of *TH* and *BH* images to the original image. By the difference (*TH* – *BH*) only details brighter than surrounding and smaller than structuring element are strongly emphasized while background tissue is highly suppressed. In fact, the gray level of previously emphasized details is increased toward white, while the overall gray level of surrounding tissue is decreased toward black. Furthermore, if we add an original image to this difference, enhanced details become even brighter than surrounding. Consequently, the enhancement of bright details smaller than the structuring element is reinforced, and uneven background (surrounding tissue texture) is highly equalized, almost regardless of overall surrounding brightness, as shown in [17].

The second method is based on the multifractal (MF) analysis. MF approach is an efficient way for quantitative description of complex structures, objects and phenomena, such as clouds, the structure of nervous system, coast-line structures, movements in global market, etc. Such objects and phenomena exhibit interesting property known as *self-similar* or *fractal* property: a structure is assumed as made of parts similar to the whole, exactly or statistically. We can distinguish two main groups of fractal structures: artificial and natural. Artificially generated fractal structures are commonly known as *deterministic* (or, *mathematical*) *fractals*[18],[19]. These structures are generated by using exact rules and they are characterized by exactly the same fractal dimension in whole scales, thus they are referred as *monofractals*.

Instead, a variety of natural objects may also exhibit self-similarity but only in some statistical sense. These structures are known as *random fractals*. The fractal dimension of such structures varies with the observed scale, thus they are referred as *multifractals*[19]. Fractal and multifractal properties of observed structure can be quantitatively described in several ways, as reported in [19-22]. After determining local regularity of the structure, for instance, through the quantity known as *Hölder exponent*, α, we can calculate the distribution of this quantity, which is known as the *multifractal spectrum*, *f*(α). The MF spectrum describes the *global regularity* of observed structure.

The MF analysis permits us to describe signal/structure features both from local and global points of view. For instance, high values of Hölder exponent α denote high local changes, and opposite for low α. Regarding to the MF spectrum *f*(α), its low values denote rare events – isolated parts in the whole structure having particular value of α, and opposite for high *f*(α). Moreover, the MF analysis may be performed in an inverse way: find parts in the signal/structure having particular values of α or *f*(α). This kind of processing may be called as an *inverseMF* (IMF) analysis. Brief description of the IMF is as follows. For given image *I*, sized *M*x*N* pixels, we can create an ‘α-image’ – a matrix of the same size *M*x*N* but filled by values of α(*i*,*j*) with one-by-one correspondence with image pixels *I*(*i*,*j*), *i*=1,2...,*M*; *j*=1,2,...,*N*. From the α matrix, the MF spectrum *f*(α), also in a matrix form, *f*(*i*,*j*)=*f*(α(*i*,*j*)), may be determined. From once created α and *f*(α) matrices (images), we can select desired range of values α and/or *f*(α), extracting in this way image parts characterized just by these multifractal values [21-24]. For instance, normal human tissue is characterized by high degree of *self-similarity*[19], while the tissue anomalies may be considered as structural “defects”, i.e., as deviations from global regularity of the background. This method is applied successfully for enhancing and detecting microcalcifications in digital mammograms [16]. Moreover, the efficiency of this method was recognized by Levy Vehel, from INRIA, France, and our program was embedded in their software package FracLab as an additional tool [25].

Regarding to FISH images, colored dots can be assumed as parts characterized by high values of Hölder exponent α (high local changes) and low values of its distribution *f*(α) (rare events, in global sense). Under these assumption, by applying IMF we can extract colored dots from nuclei background.

## Results

The two methods for detecting colored dots within segmented nuclei in FISH images: mathematical morphology (MM) method and inverse multifractal (IMF), described briefly in previous section, are tested on FISH images obtained from the Molecular Biology Lab, Department of Pathology, Wielkopolska Cancer Center, Poznan, POLAND. FISH images are evaluated by skilled pathologist and tested by proposed methods. Results for eight characteristic cases are displayed in Table 1. First four cases are labeled by physician as amplified and the rest are normal (non-amplified). As indicated in Table 1, objective methods, particularly IMF method, are promising but need improvements. The IMF method is better than MM: for all amplified cases the HER2/neu status was greater than 2.0, and for non-amplified cases was less than 1.8, except for the case 5. By using MM method results are less accurate – for amplified cases 3 and 4 the HER2/neu status was 1.71 and 1.81, while for non-amplified case 5 the value was even 5.14! By additional inspection it was found that this image was obtained without DAPI, Fig. 2. Since the nuclei segmentation was performed under the assumption of blue channel, for this image our methods counted red dots out from nuclei.

**Table 1:** The HER2/neu status calculated by applying MM and IMF methods.

Case	Diagnosis	HER2/neu status (MM)	HER2/neu status (IMF)	Remark
1	Amplified	1.96	2.23	
2	Amplified	1.94	2.22	
3	Amplified	1.71	2.14	
4	Amplified	1.81	2.02	
5	Non-amplified	5.14	2.71	Without DAPI
6	Non-amplified	0.79	0.88	
7	Non-amplified	0.26	1.12	
8	Non-amplified	0.27	1.15	

**Figure 3:**
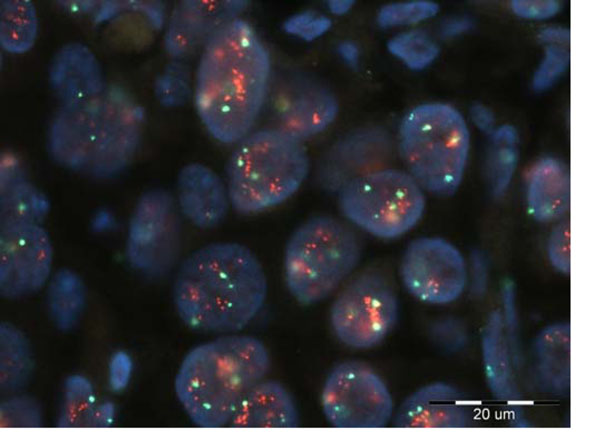
Case 1 of analyzed FISH images. Red dots are highly overlapped.

General remark regarding proposed methods is that additional post-processing is necessary, particularly for red channel. Namely, red dots are overlapped, as depicted in Fig. 3 (case 1 in our analysis) and their separation needs more sophistical processing, which will be considered in our future research.

## Conclusion

In this paper the two methods for detecting colored dots within segmented nuclei in FISH images are considered: the method based on the mathematical morphology and the inverse multifractal method. Initial results are promising but need further improvements which will be considered in our future work.

## Competing interests

The authors declare that they have no competing interests.
